# Serum carotenoids modify the association between *porphyromonas gingivalis* IgG and mortality: a prospective cohort study with 25.8 years of follow-up

**DOI:** 10.1186/s40795-026-01420-2

**Published:** 2026-07-06

**Authors:** Weihong Qiu, Lijun Zheng, Yingying Yu, Zhiquan Zhang, Zhenzhen Li, Zihan Zhang, Heng He

**Affiliations:** 1 Department of Epidemiology and Health Statistics, Fujian Provincial Key Laboratory of Environment Factors and Cancer, School of Public Health, Fujian Medical University, Fuzhou, 350122 China; 2 NHC Key Laboratory of Etiological Epidemiology of Chronic Diseases With High Incidence in Fujian-Taiwan Area (Co-Construction), Fujian Medical University, Fuzhou, 350122 China

**Keywords:** Serum carotenoids, Serum *P. gingivalis* IgG, Mortality, Cohort study

## Abstract

**Background:**

*Porphyromonas gingivalis* (*P. gingivalis*) is a vital pathogen that can increase mortality. However, whether serum carotenoids, commonly known antioxidants, modify the association of serum *P. gingivalis* immunoglobulin G (IgG) antibody levels with mortality remains uncertain. We aimed to explore the modification of this association by serum carotenoids.

**Methods:**

The associations of serum *P. gingivalis* IgG levels with mortality among 7,043 participants from the Third U.S. National Health and Nutrition Examination Survey were assessed. The modifications of serum carotenoids on the associations were also examined. We used the recommended sampling weights to account for the complex survey design. Surveycox proportional hazards regression models were used to calculate hazard ratios (HRs) and 95% confidence intervals (CIs) for all-cause and specific-cause mortality.

**Results:**

We identified 2,646 deaths during a median follow-up period of 25.8 years. Compared with participants with low serum *P. gingivalis* IgG levels (< 69 EU), those with high levels (≥ 69 EU) were associated with an HR (95% CI) of 1.10 (1.04–1.16) for all-cause mortality. Similar results were found in mortality analyses for cardiovascular disease, cerebrovascular disease, and diabetes mellitus (all* P* < 0.05). Notably, significant interactions were detected between the serum α-carotene, β-carotene, or lutein/zeaxanthin concentrations and the serum *P. gingivalis* IgG levles on all-cause mortality (all* P*_int_ < 0.05). Compared with high *P. gingivalis* IgG participants with low carotenoids, low *P. gingivalis* IgG participants with high carotenoids had the lowest risk of all-cause mortality, with HR of 0.74 (0.68–0.80) for α-carotene, 0.78 (0.72–0.86) for β-carotene, 0.70 (0.64–0.76) for β-cryptoxanthin, 0.79 (0.73–0.85) for lutein/zeaxanthin and 0.64 (0.59–0.69) for lycopene, respectively.

**Conclusion:**

Serum carotenoids mitigate the associations between serum *P. gingivalis* IgG levels and mortality.

**Supplementary Information:**

The online version contains supplementary material available at https://doi.org/10.1186/s40795-026-01420-2.

## Introduction

Diverse and abundant microbial communities are present in the human oral cavity, and play a pivotal role in maintaining the delicate balance of the local oral environment. When the homeostasis of these microbial communities is disrupted, a wide array of pathological reactions can be triggered, ranging from periodontal diseases to cardiovascular and metabolic ailments, neurologic disorders, gastrointestinal issues, and even cancers [1]. Among the numerous microbiota, *Porphyromonas gingivalis* (*P. gingivalis*) stands out as a particularly troublesome and widely recognized gram-negative microbe [2]. This microorganism is one of the most severe pathogens associated with periodontal infections, posing a significant threat to oral health [3].

In most individuals afflicted with oral diseases, there is a marked elevation in serum immunoglobulin G (IgG) antibodies directed against *P. gingivalis* (*P. gingivalis* IgG) [4]. Serum *P. gingivalis* IgG titers persist at elevated levels for up to 15 years [5], which is indicative of both past and ongoing periodontal infections. Consequently, *P. gingivalis* IgG serves as a cumulative measure of periodontal infection over time [6]. *P. gingivalis* IgG has been linked to various health outcomes, including all-cause mortality [7], cardiovascular diseases (CVD) [8–10], rheumatoid arthritis [11], chronic kidney disease [12], cognitive impairment [13, 14], and various types of cancers [15, 16]. In previous research conducted by Damian et al., a nonlinear correlation was identified between serum *P*. *gingivalis* IgG and mortality via the data from the Third US National Health and Nutrition Examination Survey (NHANES III), which followed up to 2006 [7]. We further utilized NHANES III data followed up to 2019 to explore the associations between *P*. *gingivalis* and all-cause and specific-cause mortality.

Carotenoids are a widely recognized class of antioxidants and immunomodulatory compounds, with more than 700 unique varieties identified to date. Among them, α-carotene, β-carotene, β-cryptoxanthin, lycopene, lutein, and zeaxanthin account for more than 95% of the total carotenoids [17, 18]. Researches have shown that carotenoids can effectively prevent diseases such as periodontitis, indicating their potential to regulate the oral microbiota, including inflammation caused by *P*. *gingivalis* [19, 20]. Given the strong correlation between periodontitis and mortality, coupled with the antioxidant and anti-inflammatory functions of carotenoids, we hypothesize that serum carotenoids may modify the association between serum antibodies against *P. gingivalis* and all-cause and specific-cause mortality.

Therefore, in this study, we utilized data from NHANES III to further evaluate the associations between serum *P*. *gingivalis* IgG and mortality, with follow-up extending until 2019. Additionally, we examined the modified associations of carotenoids on these associations, providing valuable insights and evidence for the prevention of death linked to the oral microbiota.

## Methods

### Study population

The study participants were from NHANES III, which was described previously [21]. In short, NHANES III was conducted by the National Center for Health Statistics (NCHS) between 1988 and 1994 in two phases in which the civilian noninstitutionalized U.S. population was sampled to provide a multidimensional overview of physical health status and aspects of functional health status. After excluding participants with missing information on serum carotenoids, serum *P. gingivalis* IgG, mortality status, and those who were pregnant or already had cancer at the baseline, a total of 7,043 participants ≥ 20 years of age from NHANES III were included in this study (Figure S1). Patients or the public were not involved in the design, conduct, reporting, or dissemination plans of our research.

### Covariate collection

Each participant’s baseline demographic characteristics, disease and medication history, and lifestyle were collected via face-to-face questionnaires, including sex (male or female), age groups (20–39,40–59, ≥ 60 years), race-ethnicity (non-Hispanic White, non-Hispanic Black, Mexican American, other), education (less than junior high school, junior high school, high school, college or above), annual total income of the family (< $20,000, ≥ $20,000), smoking status (current smokers, former smokers, never smokers), drinking status (≥ 12 drinks in the past 12 months, < 12 drinks in the past 12 months), exercise status (more active, less active, about the same), pregnancy status (yes or no), and self-reported cancer status (yes or no). Body mass index (BMI) was calculated by dividing weight in kilograms (kg) by height in meters squared (m^2^) and was divided into four groups (< 18.5, 18.5–24.9, 25–29.9, or > 30 kg/m^2^).

### Assessment of the serum IgG antibody against P. gingivalis

Serum *P. gingivalis* IgG levels at baseline was chosen for this study to reflect evidence of systemic exposure to this bacterium, as it is a validated periodontal pathogen [22] and has well-described periodontal pathogenicity [23]. The levels of the serum *P. gingivalis* IgG were quantified via enzyme-linked immunosorbent assay, and the antibody measurements were reported in units of IgG (EU), which has been reported in detail in the documentation of periodontal pathogens of NHANES III [24]. Based on a previous study [15], we divided the *P. gingivalis* IgG levels into three categories: low (< 69 EU), intermediate (69–119.0 EU), and high (> 119.0 EU).

### Assessment of serum carotenoids

In this study, we included six serum carotenoids represented by five serum measures including α-carotene, β-carotene, β-cryptoxanthin, lutein/zeaxanthin, and lycopene at baseline which are available from NHANES III. Serum concentrations of these carotenoids were measured via high-performance liquid chromatography (HPLC) in NHANES III. The laboratory procedures and quality control methods used for the serum carotenoids measurements were described in detail previously [25, 26]. Serum carotenoids concentrations were divided into low or high groups based on the median.

### Ascertainment of mortality

Data on all-cause and specific-cause of death were obtained from the public-use data files available from the National Center for Health Statistics (NCHS). Vital status was determined by the National Death Index (NDI), and the cause of death was determined by the basic cause of death listed on the death certificate. Vital status and the cause of death were linked to data from NHANES III for further analysis. The International Classification of Diseases (ICD), 9th Revision (ICD-9) was used for coding all causes of death occurring before 1999, while the ICD-10 was used for coding deaths after 1998. The NCHS created a UCOD_113 variable to recode all deaths into comparable underlying causes of death groups, to assist researchers in analyzing specific death periods throughout the entire survey. We selected all causes of death and several major causes of death, including CVD, cancer, cerebrovascular diseases (CeVD), and diabetes mellitus (DM) as outcomes. For each study participant, follow-up extended from baseline to the date of death or end of follow-up (31 December, 2019), whichever occurred first.

### Statistical analysis

Baseline demographic characteristics were described according to serum *P. gingivalis* IgG levels (low [< 69 EU], intermediate [69–119.0 EU], and high [> 119.0 EU]). The demographic characteristics of the different *P. gingivalis* IgG groups were compared via the chi-square test (categorical variables) or one-way analysis of variance (continuous variables). Person-years were calculated from baseline to the date of death or the end of follow-up (31 December, 2019), whichever occurred first.

We used the recommended sampling weights to account for the NHANES' complex survey design. Specifically, we applied the MEC- and home-examined sample final weight (WTPFHX6), with variance estimated using the pseudo-stratum (SDPSTRA6) and pseudo-PSU (SDPPSU6) variables as recommended by NCHS. Two variable-adjusted Surveycox proportional hazards regression models were conducted to calculate hazard ratios (HRs) and 95% confidence intervals (CIs) for all-cause and specific-cause mortality in relation to serum *P. gingivalis* IgG levels. Model 1 was adjusted for sex and age groups. Model 2 was additionally adjusted for potential confounders: race-ethnicity, education, income, smoking status, drinking status, exercise, and BMI groups. The proportional hazards assumption was examined by creating a product term of follow-up time and exposure, and we found no significant deviation from the assumption.

We further conducted stratified analyses by groups of serum carotenoids (low or high) to investigate associations of the serum *P. gingivalis* IgG levels with all-cause and specific-cause mortality. To assess multiplicative interaction, we included in the Cox model the main terms for serum *P. gingivalis* IgG (dichotomized as low [< 69 EU] vs. high [≥ 69 EU]) and serum carotenoids (low vs. high), along with their product term. Both dichotomous variables were coded as 1 and 2, and the product term was calculated as the simple multiplication of the two. The Wald test for the coefficient of the product term was used to evaluate interaction on the multiplicative scale, and the corresponding hazard ratio (HR) with its 95% confidence interval (CI) is reported as the measure of interaction. To analyze the joint associations of *P. gingivalis* IgG concentration and carotenoids concentration on mortality, we divided the participants into four groups according to *P. gingivalis* IgG (< 69 EU vs. ≥ 69 EU) and the serum carotenoids (low vs. high) and estimated HRs (95% CIs) of mortality in different groups compared with those with serum *P. gingivalis* IgG ≥ 69 EU and low serum carotenes.

We also performed sensitivity analyses using tertiles of the serum *P. gingivalis* IgG distribution. In addition, the high-exposure group was redefined as the top two tertiles combined, and the stratified analyses, interaction analysis as well as the joint association models were repeated using the alternative cutoff.

Taking into account the complex survey sampling of NHANES, the survival probabilities for all-cause mortality and specific cause mortality were calculated and plotted via the R packages “survey” and “jskm”, respectively. Restricted cubic spline (RCS) curves for sampling weights were depicted to show the nonlinear relationships between serum *P. gingivalis* IgG levels and mortality. All analyses were performed via SAS 9.4 (SAS Institute Inc., Cary, NC, USA) and R 4.0.2 (R Core Team, Vienna, Austria), and a two-sided *P* < 0.05 was considered statistically significant.

## Results

### Descriptive statistics

A total of 7,043 (3,127 males; 44.4%) participants were included, and their characteristics according to the groups of serum *P. gingivalis* IgG (< 69 EU, 69–119 EU, and > 119 EU) are displayed in Table 1. Participants with higher serum *P. gingivalis* IgG levels tended to be older, non-Hispanic Black and Mexican American, non-current smokers, who had attended less than junior high school. These participants also tended to have a higher BMI, a lower income, were less likely to be physically active, and were less likely to drink alcohol (all *P* < 0.05).

**Table 1:** Baseline characteristics of participants according to serum *P. gingivalis* IgG

Characteristics	Total	Serum *Porphyromonas gingivalis* IgG (EU)			*P* value^a^
		** < 69**	**69–119**	** > 119**	
N	7043	1948	3384	1711	
Follow-up years, median (IQR)	25.83 (17.75–27.00)	25.92 (19.92–27.08)	25.92 (17.92–27.00)	25.67 (15.92–26.92)	0.001
Gender (%)					0.002
Male	3127 (44.4)	805 (41.3)	1518 (44.9)	804 (47.0)	
Female	3916 (55.6)	1143 (58.7)	1866 (55.1)	907 (53.0)	
Age (years), mean (SD)	46.48 (18.56)	45.20 (19.56)	46.12 (18.43)	48.64 (17.44)	< 0.001
Age groups (years) (%)					< 0.001
20–39	3099 (44.0)	960 (49.3)	1513 (44.7)	626 (36.6)	
40–59	1929 (27.4)	455 (23.4)	940 (27.8)	534 (31.2)	
≥ 60	2015 (28.6)	533 (27.4)	931 (27.5)	551 (32.2)	
Race-ethnicity (%)					< 0.001
Non-Hispanic white	2536 (36.0)	1159 (59.5)	1089 (32.2)	288 (16.8)	
Non-Hispanic black	2177 (30.9)	351 (18.0)	1063 (31.4)	763 (44.6)	
Mexican American	1980 (28.1)	386 (19.8)	1026 (30.3)	568 (33.2)	
Other	350 (5.0)	52 (2.7)	206 (6.1)	92 (5.4)	
Education (%)					< 0.001
Less than junior high school	1654 (23.5)	318 (16.3)	839 (24.8)	497 (29.0)	
Junior high school	1100 (15.6)	264 (13.6)	571 (16.9)	265 (15.5)	
High school	2218 (31.5)	629 (32.3)	1062 (31.4)	527 (30.8)	
College or above	2027 (28.8)	727 (37.3)	895 (26.4)	405 (23.7)	
Missing	44 (0.6)	10 (0.5)	17 (0.5)	17 (1.0)	
Income (%)					< 0.001
< $20,000	3343 (47.5)	756 (38.8)	1635 (48.3)	952 (55.6)	
≥ $20,000	3562 (50.6)	1160 (59.5)	1674 (49.5)	728 (42.5)	
Missing	138 (2.0)	32 (1.6)	75 (2.2)	31 (1.8)	
Smoking status (%)					< 0.001
Current smokers	1767 (25.1)	535 (27.5)	897 (26.5)	335 (19.6)	
Former smokers	1588 (22.5)	426 (21.9)	773 (22.8)	389 (22.7)	
Never smokers	3688 (52.4)	987 (50.7)	1714 (50.7)	987 (57.7)	
Drinking status (%)					0.001
≥ 12 drinks in the past 12 months	5548 (78.8)	1581 (81.2)	2670 (78.9)	1297 (75.8)	
< 12 drinks in the past 12 months	1318 (18.7)	322 (16.5)	639 (18.9)	357 (20.9)	
Missing	177 (2.5)	45 (2.3)	75 (2.2)	57 (3.3)	
Exercise (%)					0.15
More active	2176 (30.9)	617 (31.7)	1060 (31.3)	499 (29.2)	
Less active	1556 (22.1)	408 (20.9)	734 (21.7)	414 (24.2)	
About the same	3217 (45.7)	893 (45.8)	1552 (45.9)	772 (45.1)	
Missing	94 (1.3)	30 (1.5)	38 (1.1)	26 (1.5)	
BMI groups					< 0.001
< 18.5	136 (1.9)	40 (2.1)	70 (2.1)	26 (1.5)	
18.5 ~ < 25	2457 (34.9)	792 (40.7)	1182 (34.9)	483 (28.2)	
25 ~ < 30	2455 (34.9)	653 (33.5)	1186 (35.0)	616 (36.0)	
≥ 30	1988 (28.2)	463 (23.8)	942 (27.8)	583 (34.1)	
Missing	7 (0.1)	0 (0.0)	4 (0.1)	3 (0.2)	
α-carotene (nmol/L), median (IQR)	70 (40–110)	70 (40–110)	70 (40–110)	60 (20–90)	0.001
β-carotene (nmol/L), median (IQR)	260 (150–450)	280 (170–470)	260 (150–450)	260 (150–430)	0.07
β-cryptoxanthin (nmol/L), median (IQR)	160 (110–240)	140 (110–220)	160 (110–250)	160 (110–250)	< 0.001
Lutein/zeaxanthin (nmol/L), median (IQR)	350 (260–470)	325 (250–440)	350 (260–490)	370 (260–510)	< 0.001
Lycopene (nmol/L), median (IQR)	370 (260–540)	410 (280–580)	370 (260–520)	350 (240–500)	< 0.001

*Abbreviation*: *SD* standard deviation, *95% CI* 95% confidence interval, *BMI* Body mass index, *IQR* Interquartile Range

^a^*P*-values were based on Chi-square test (categorical variables) or one-way analysis of variance (normal continues variables) or Kruskal–Wallis Rank Sum Test (nonnormal continues variables)

### Associations of serum *P. gingivalis* IgG levels with mortality

**Fig. 1:**
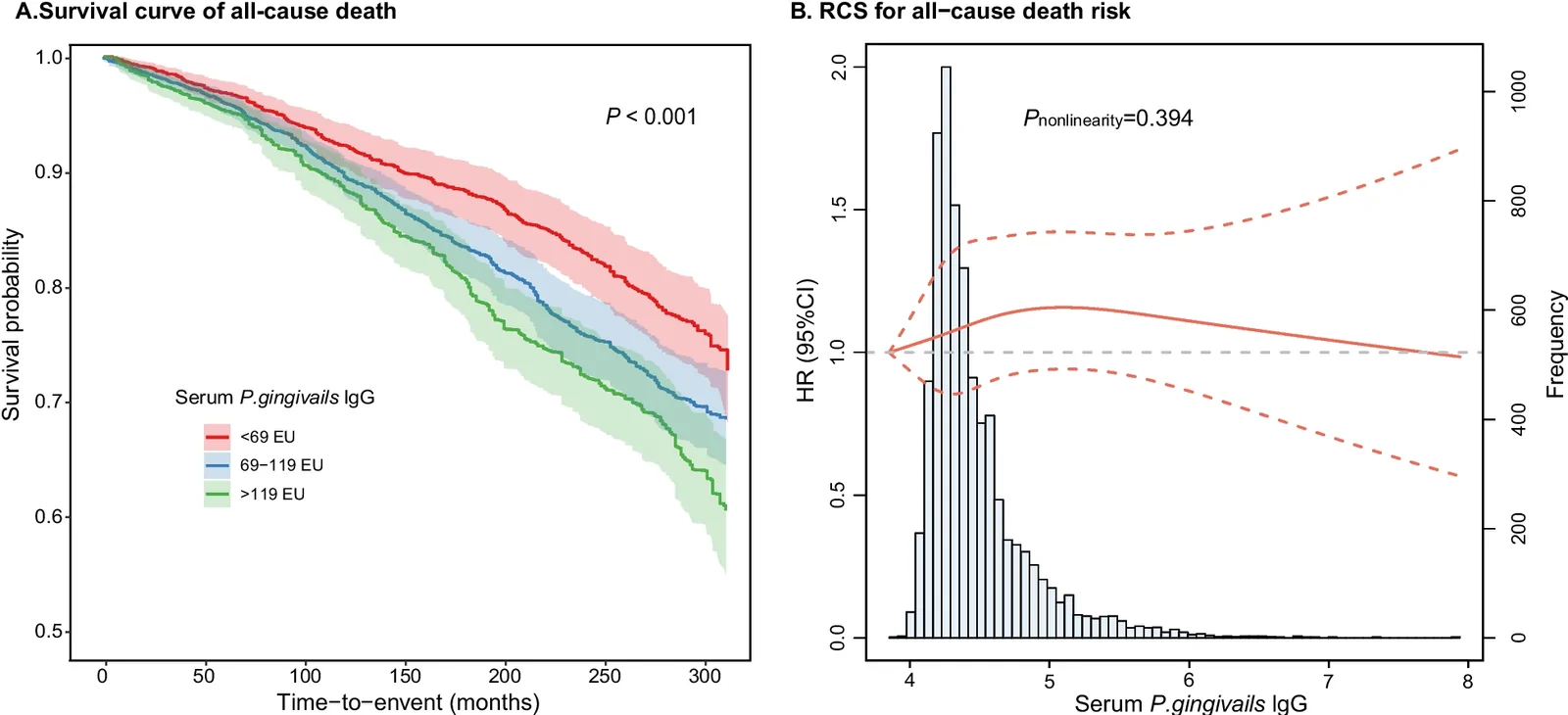
The survival probability of all-cause mortality by serum *P. gingivalis* IgG groups and the dose–response associations between serum *P. gingivalis* IgG levels and all-cause mortality. **A** survival curve; **B** restricted cubic spline. Abbreviation: *P. gingivalis*, Porphyromonas gingivalis; HR, hazard ratio; CI, confidence interval; IgG, immunoglobulin G. The restricted cubic spline model was adjusted by sex (males or females), age (20–39, 40–59, ≥ 60 years), race-ethnicity (non-Hispanic white, non-Hispanic black, Mexican American and other), education (less than junior high school, junior high school, high school, college or above), income (< $20,000, ≥ $20,000), smoking status (current smokers, former smokers, never smokers), drinking status (≥ 12 drinks in the past 12 months, < 12 drinks in the past 12 months), exercise (more active, less active, about the same), body mass index (< 18.5, 18.5–24.9, 25–29.9, ≥ 30 kg/m.^2^)

The survival probability of participants with all-cause mortality was highest among those with low serum *P. gingivalis* IgG levels (< 69 EU), followed by those with intermediate (69–119 EU) and high *P. gingivalis* IgG levels (> 119 EU) (*P* < 0.001; Fig. 1A). Similar results were found for CVD, cancer, and CeVD mortality (Figure S2). The dose–response association results revealed that serum *P. gingivalis* IgG levels were linearly associated with the risk of all-cause, CVD, cancer, CeVD, and DM mortality (all *P*_nonlinearity_ > 0.05; Fig. 1B and Figure S3).

The associations of classified *P. gingivalis* IgG with all-cause and specific mortality are shown in Table 2. A total of 2,646 all-cause deaths, 761 CVD deaths, 553 cancer deaths, 132 CeVD deaths, and 111 DM deaths were identified during a median follow-up period of 25.8 years. Compared with low *P. gingivalis* IgG levels (< 69 EU), those with intermediate (69–119 EU) and high *P. gingivalis* IgG levels (> 119 EU) were associated with multivariable-adjusted HRs (95% CIs) of 1.10 (1.04 to 1.16) and 1.11 (1.05 to 1.17) for all-cause mortality (*P*_trend_ < 0.001); 1.23 (1.08 to 1.39) and 1.22 (1.03 to 1.44) for CVD mortality (*P*_trend_ = 0.010); 1.06 (0.91 to 1.23) and 1.22 (1.09 to 1.37) for cancer mortality (*P*_trend_ = 0.007); 1.31 (1.13 to 1.53) and 1.25 (1.04 to 1.49) for CeVD mortality (*P*_trend_ = 0.012); and 2.81 (2.54 to 3.12) and 2.18 (1.85 to 2.58) for DM mortality (*P*_trend_ < 0.001), respectively. Additionally, compared with low *P. gingivalis* IgG levels (< 69 EU), those with high *P. gingivalis* IgG levels (≥ 69 EU) have a higher risk of all-cause mortality (HR, 1.10; 95% CI, 1.05 to 1.16), CVD mortality (HR, 1.23; 95% CI, 1.09 to 1.38), CeVD mortality (HR, 1.30; 95% CI, 1.11 to 1.51), and DM mortality (HR, 2.66; 95% CI, 2.40 to 2.96), respectively. Sensitivity analyses using tertile-based cutoffs yielded generally similar results (Table S1).

**Table 2:** Hazard ratios (95% CIs) of mortality in relation to serum *P. gingivalis* IgG

	Serum *Porphyromonas gingivalis* IgG				
	< 69 EU	69–119 EU	> 119 EU	*P* for trend	69 + EU
All-cause mortality					
Cases/person-years	681/43,037	1238/73,783	727/36,267		1965/110,050
Age and gender-adjusted HR (95% CI)	1.00 (ref)	1.15 (1.10–1.20)	1.15 (1.05–1.26)	0.002	1.15 (1.09–1.21)
Multivariable-adjusted HR (95% CI)^*^	1.00 (ref)	1.10 (1.04–1.16)	1.11 (1.05–1.17)	< 0.001	1.10 (1.05–1.16)
CVD mortality					
Mortality/person-years	200/36,132	350/61,714	211/28,845		561/90,558
Age and gender-adjusted HR (95% CI)	1.00 (ref)	1.25 (1.10–1.43)	1.15 (1.01–1.32)	0.02	1.23 (1.08–1.39)
Multivariable-adjusted HR (95% CI)^*^	1.00 (ref)	1.23 (1.08–1.39)	1.22 (1.03–1.44)	0.010	1.23 (1.09–1.38)
Cancer mortality					
Mortality/person-years	134/35,640	256/60,893	163/28,446		419/89,339
Age and gender-adjusted HR (95% CI)	1.00 (ref)	1.03 (0.96–1.10)	1.15 (0.99–1.35)	0.07	1.06 (0.99–1.14)
Multivariable-adjusted HR (95% CI)^*^	1.00 (ref)	1.06 (0.91–1.23)	1.22 (1.09–1.37)	0.007	1.10 (0.97–1.25)
CeVD mortality					
Mortality/person-years	48/34,474	58/58,116	26/26,639		84/84,755
Age and gender-adjusted HR (95% CI)	1.00 (ref)	1.22 (1.07–1.40)	1.20 (0.98–1.47)	0.047	1.22 (1.05–1.42)
Multivariable-adjusted HR (95% CI)^*^	1.00 (ref)	1.31 (1.13–1.53)	1.25 (1.04–1.49)	0.01	1.30 (1.11–1.51)
DM mortality					
Mortality/person-years	16/33,946	60/58,191	35/26,764		95/84,955
Age and gender-adjusted HR (95% CI)	1.00 (ref)	2.55 (2.38–2.74)	2.30 (1.97–2.69)	< 0.001	2.49 (2.30–2.69)
Multivariable-adjusted HR (95% CI)^*^	1.00 (ref)	2.81 (2.54–3.12)	2.18 (1.85–2.58)	< 0.001	2.66 (2.40–2.96)

*Abbreviations*: *HR* hazard ratio, *CI* confidence interval, *CVD* cardiovascular disease, *CeVD* cerebrovascular disease, *DM* diabetes mellitus

^*^The cox proportional hazard models were adjusted by sex (males or females), age (20–39,40–59, ≥ 60 years), race-ethnicity (non-Hispanic white, non-Hispanic black, Mexican American and other), education (less than junior high school, junior high school, high school, college or above), income (< $20,000, ≥ $20,000), smoking status (current smokers, former smokers, never smokers), drinking status (≥ 12 drinks in the past 12 months, < 12 drinks in the past 12 months), exercise (more active, less active, about the same), body mass index (< 18.5, 18.5–24.9, 25–29.9, ≥ 30 kg/m^2^)

### Interactions of serum carotenoids and *P. gingivalis* IgG levels on mortality

**Fig. 2:**
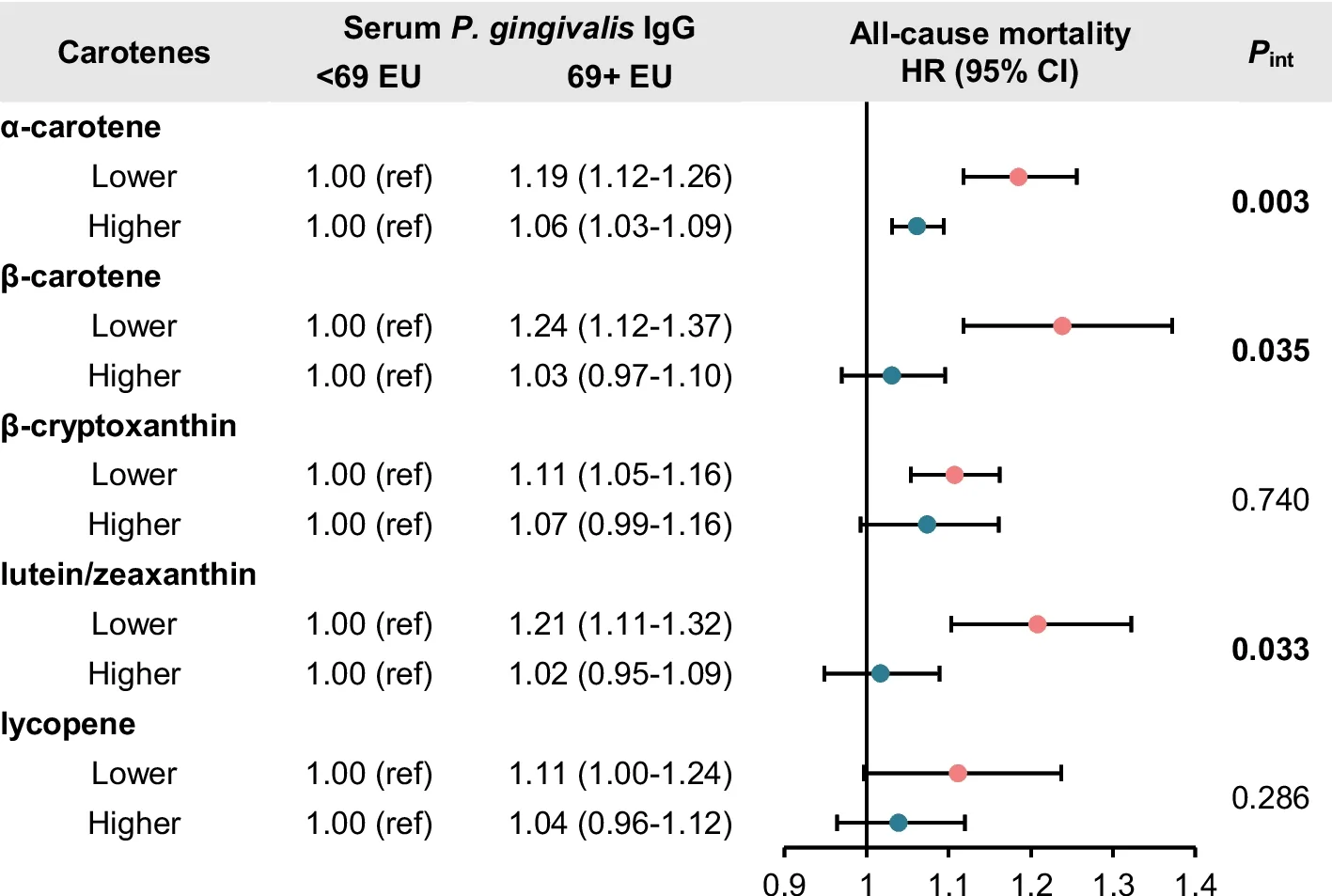
The interactions between serum carotenoids and serum *P. gingivalis* IgG levels on all-cause mortality. Abbreviation: *P. gingivalis*, Porphyromonas gingivalis; IgG, immunoglobulin G; CI, confidence interval. The cox proportional hazard models were adjusted by sex (males or females), age (20–39, 40–59, ≥ 60 years), race-ethnicity (non-Hispanic white, non-Hispanic black, Mexican American and other), education (less than junior high school, junior high school, high school, college or above), income (< $20,000, ≥ $20,000), smoking status (current smokers, former smokers, never smokers), drinking status (≥ 12 drinks in the past 12 months, < 12 drinks in the past 12 months), exercise (more active, less active, about the same), body mass index (< 18.5, 18.5–24.9, 25–29.9, ≥ 30 kg/m.^2^)

Significant interactions were found between serum α-carotene, β-carotene, or lutein/zeaxanthin and *P. gingivalis* IgG on all-cause mortality (all* P*_int_ < 0.05) (Fig. 2). Higher *P. gingivalis* IgG levels were associated with increased risks of all-cause mortality among individuals in various serum carotenoids subgroups, whereas the associations were weaker among those in the higher serum carotenoids subgroups (Fig. 2). Specifically, compared with low serum *P. gingivalis* IgG levels (< 69 EU), high *P. gingivalis* IgG levels (≥ 69 EU) were associated with multivariable-adjusted HRs (95% CIs) of 1.19 (1.12 to 1.26) in the lower α-carotene group and 1.06 (1.03 to 1.09) in the higher α-carotene group (*P*_int_ = 0.003), 1.24 (1.12 to 1.37) in the lower β-carotene group and 1.03 (0.97 to 1.10) in the higher β-carotene group (*P*_int_ = 0.04), and 1.21 (1.11 to 1.32) in the lower lutein/zeaxanthin group and 1.02 (0.95 to 1.09) in the higher lutein/zeaxanthin group (*P*_int_ = 0.03) (Fig. 2).

In addition, interaction analyses were also conducted for CVD, cancer, CeVD, and DM mortality, the results of which are shown in Figure S4-S7, respectively. Similar results were found for CeVD and DM mortality, except for β-cryptoxanthin for CeVD mortality (all* P*_int_ < 0.05) (Figure S6-S7). We found significant interactions of serum β-cryptoxanthin and serum *P. gingivalis* IgG levels on the risk of cancer and CeVD mortality (both* P*_int_ < 0.05), whereas the associations were weaker among those from the lower serum β-cryptoxanthin subgroups when stratified by serum β-cryptoxanthin (Figure S5-S6).

In sensitivity analyses using tertiles to categorize serum carotenoid levels (Table S2), the interaction patterns for all-cause, CVD, CeVD, and DM mortality remained generally similar. In the primary analysis for cancer mortality, no significant interaction was observed between serum levels of α-carotene, β-carotene and *P. gingivalis* IgG. However, in the sensitivity analysis, a significant interaction emerged (Table S2).

### Joint analyses of serum carotenoid and *P. gingivalis* IgG levels with mortality

**Fig. 3:**
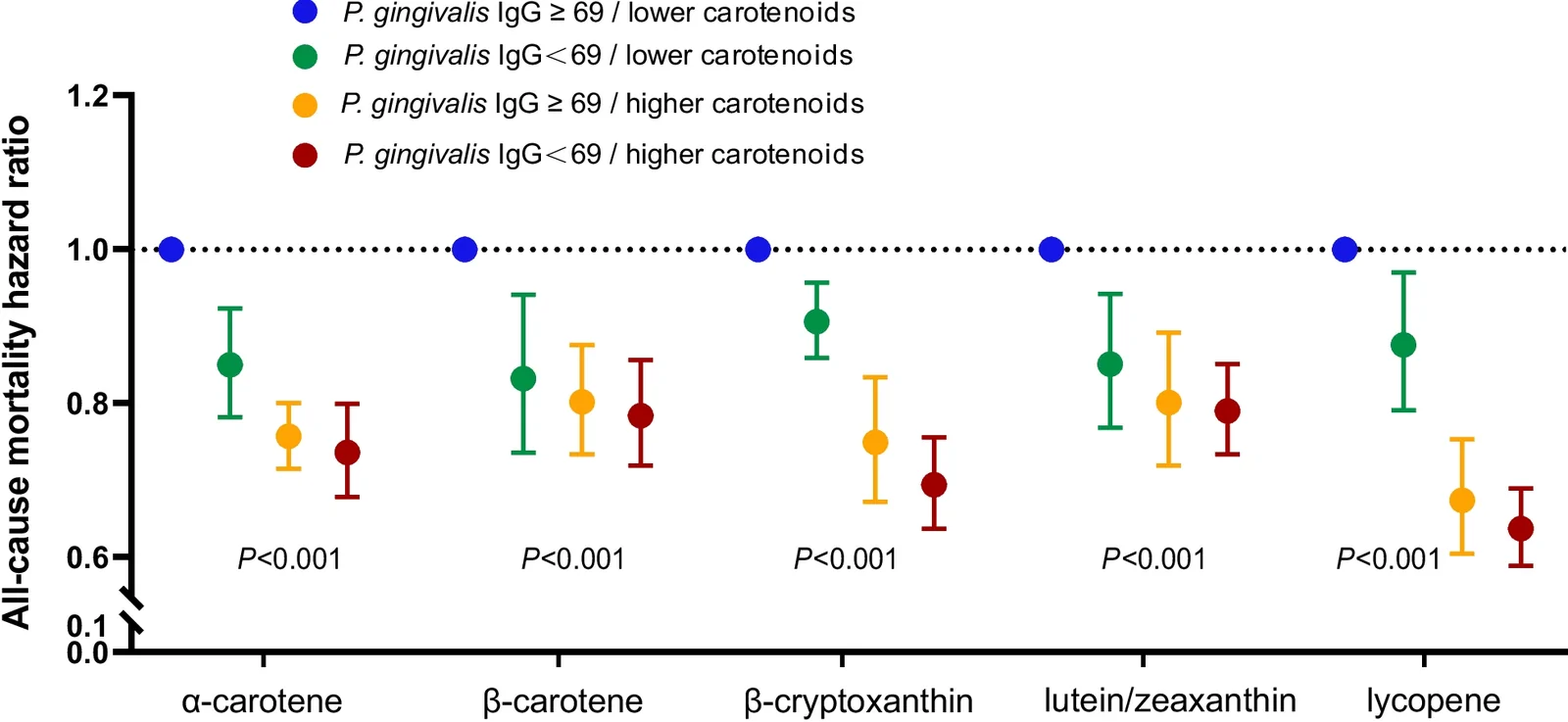
The joint associations of serum carotenoids and serum *P. gingivalis* IgG levels on all-cause mortality. Abbreviation: *P. gingivalis*, Porphyromonas gingivalis; IgG, immunoglobulin G. The cox proportional hazard models were adjusted by sex (males or females), age (20–39, 40–59, ≥ 60 years), race-ethnicity (non-Hispanic white, non-Hispanic black, Mexican American and other), education (less than junior high school, junior high school, high school, college or above), income (< $20,000, ≥ $20,000), smoking status (current smokers, former smokers, never smokers), drinking status (≥ 12 drinks in the past 12 months, < 12 drinks in the past 12 months), exercise (more active, less active, about the same), body mass index (< 18.5, 18.5–24.9, 25–29.9, ≥ 30 kg/m.^2^)

We observed significant joint associations of serum carotenoids and *P. gingivalis* IgG on all-cause mortality in an exposure–response manner (Fig. 3). Specifically, compared with participants with higher serum *P. gingivalis* IgG levels (≥ 69 EU) and lower serum carotenoids, participants with lower serum *P. gingivalis* IgG levels (< 69 EU) and higher serum carotenoids presented the lowest risk of all-cause mortality, with HRs (95% CIs) of 0.74 (0.68–0.80) for α-carotene, 0.78 (0.72–0.86) for β-carotene, 0.69 (0.64–0.76) for β-cryptoxanthin, 0.79 (0.73–0.85) for lutein/zeaxanthin and 0.64 (0.59–0.69) for lycopene, (Fig. 3). The results of the joint analyses of serum carotenoids and serum *P. gingivalis* IgG levels with CVD and cancer mortality were similar, but not for CeVD and DM (Figure S8). Sensitivity analyses using tertile-based cutoffs gave similar findings for joint analyses(Table S3).

## Discussion

In the national population-based prospective cohort study conducted in NHANES III, we observed that serum *P. gingivalis* IgG levels were positively associated with all-cause mortality. We also found that the association was significantly modified by serum α-carotene, β-carotene, and lutein/zeaxanthin, and the associations between serum *P. gingivalis* IgG levels and all-cause mortality were weaker among those with higher carotenoid levels. The lowest risks of all-cause mortality were observed in those with low *P. gingivalis* IgG levels and high carotenoids levels. In addition, we observed that serum *P. gingivalis* IgG levels were positively associated with CVD, cancer, CeVD, and DM mortality. Significant interactions were found between serum carotenoid and serum *P. gingivalis* IgG levels with respect to CeVD and DM mortality.

Our findings revealed a positive association between serum *P. gingivalis* IgG levels and all-cause mortality. Dose–response association analysis revealed a linear relationship between the serum *P. gingivalis* IgG concentration and the risk of all-cause mortality. However, a prospective cohort study conducted by Damian et al. in 2015 reported a nonlinear association of serum *P. gingivalis* IgG levels with mortality [7]. This difference in findings may suggest that the relationship between *P. gingivalis* IgG levels and mortality is more complex than previously understood. Although the precise mechanisms underlying the observed positive association between the serum *P. gingivalis* IgG and the all-cause mortality remain unclear, several potential mechanisms may explain this relationship. The pathogenicity of *P. gingivalis* is primarily attributed to its ability to interfere with the host immune system, through virulence factors and cellular immune responses, which are mediated mainly by T cells. These immune modulations may influence the etiology of various systemic diseases, such as atherosclerosis, hypertension, adverse pregnancy outcomes, inflammatory bowel disease, DM, nonalcoholic fatty liver disease, rheumatoid arthritis, and Alzheimer’s disease [27]. These pathological effects may ultimately contribute to increase risks in all-cause mortality.

In addition, our study revealed that serum *P. gingivalis* IgG levels were positively associated with CVD, cancer, CeVD, and DM mortality. A previous study revealed that the *P. gingivalis* antibody titer in the serum is significantly positively correlated with the aortic valve area and Doppler velocity index [10]. Additionally, *P. gingivalis* has been shown to regulate atherosclerosis through immune pathways [28], further indicating a substantial relationship between this oral bacterium and the pathogenesis of CVD. Possible oncogenic mechanisms of *P. gingivalis* include the production of virulence factors or active metabolites, the activation of oncogenic signaling pathways, the promotion of aberrant immune responses, and the inhibition of host cell apoptosis, all of which align with our findings related to cancer mortality [29]. This study also revealed a significant link between serum *P. gingivalis* IgG levels and DM mortality, possibly because *P. gingivalis* can trigger a systemic inflammatory response, disrupt insulin signaling, and induce β-cell hypofunction and decrease [30]. These findings suggest that *P. gingivalis* infection may play a significant role in the pathogenesis and prognosis of these life-threatening conditions.

For the first time, we found that serum carotenoids significantly modified the relationship between serum *P. gingivalis* IgG levels and all-cause mortality. This discovery suggests that serum carotenoids may reduce the risk of death associated with *P. gingivalis*. Serum carotenoids are well-established composite biomarkers of fruit and vegetable intake and systemic antioxidant status [31, 32]. Their modifying associations likely reflects a direct counteraction of *P. gingivalis*-induced oxidative stress and inflammation through multiple mechanisms. Carotenoids are potent lipophilic antioxidants that may scavenge reactive oxygen species (ROS) generated during *P. gingivalis* infection and other metabolic processes [33]. At the molecular level, carotenoids activate the transcription factor nuclear factor erythroid 2-related factor 2 (Nrf2), which in turn upregulates the expression of a battery of antioxidant and cytoprotective enzymes, such as heme oxygenase-1 (HO-1) [34]. Concurrently, carotenoids are known to inhibit the activation of nuclear factor-κB (NF-κB) [34], a key pro-inflammatory transcription factor, and suppress the production of major pro-inflammatory cytokines including tumor necrosis factor (TNF), interleukin (IL)−1β, and IL-6 [35]. This finding explains the specific positive impact that carotenoids exert on the body, highlighting their potential health benefits. Similarly, vitamin D has been reported to reduce *P. gingivalis* infection by promoting autophagy, enhancing innate immunity in gingival epithelial cells, and modulating periodontal inflammation [36–39]. These findings suggest that, like carotenoids, vitamin D may also exert a protective effect against *P. gingivalis*-related mortality, although this hypothesis warrants further investigation.

The interactions between serum carotenoid and serum *P. gingivalis* IgG levels on DM are obvious both in primary analyses and sensitivity analyses. Individuals with high carotenoid levels have a reduced risk of DM mortality associated with serum *P. gingivalis* IgG levels. These results indicated that carotenoids may effectively alleviate the association of *P. gingivalis* IgG levels on DM mortality. Under high glucose conditions, β-carotene significantly inhibited the production of TNF, IL-1 and monocyte chemotactic protein-1 (MCP-1) induced by *P. gingivalis* lipopolysaccharide (LPS) in human acute monocytic leukemia cells (THP)−1 monocytes [40]. This study suggested that β-carotene may be a potential anti-inflammatory nutrient for circulating *P. gingivalis* LPS-mediated cytokine production in diabetic patients with periodontitis [40]. β-carotene may help interfere with diabetic complications by targeting intracellular NF-kB. Diabetic patients may be able to prevent or delay the development of several complications mediated by periodontitis by using appropriate dietary treatment with β-carotene [20]. For cancer mortality, the modifying associations of serum carotenoids was less consistent. No significant interaction was observed in the primary analysis, but a significant interaction emerged in the sensitivity analysis. This discrepancy may reflect differences in cut-point selection and statistical power, as well as the complexity of the tumor microenvironment and cancer heterogeneity. Further studies are needed to validate these findings.

To the best of our knowledge, this is the first prospective study to assess the interaction between serum carotenoid and serum *P. gingivalis* IgG levels on mortality. The main purpose of this study was to explore the associations between serum *P. gingivalis* IgG levels and all-cause mortality and specific-cause mortality in a national prospective cohort survey population with a median follow-up period of 25.8 years and to evaluate the modifying associations of serum carotenoids on this association. The findings revealed that carotenoids may have a protective effect on mortality associated with serum *P. gingivalis* IgG levels. Our study also has several limitations. First, while *P. gingivalis* is a well-characterized oral bacterium, serum antibody assessment is an indirect measure of oral gastrointestinal bacterial exposure on the basis of individual variable vascular exposure and the immune response. Second, serum carotenoids and *P. gingivalis* IgG were measured only at baseline, and no repeated dietary data were collected during follow-up. This single-measurement approach cannot account for temporal changes in biomarkers or diet, potentially leading to regression dilution [41] and additional exposure misclassification. Third, although baseline age was adjusted for in the Cox modles, age-related mortality risk over the long-term follow-up may still influence the survival curves. The irregularities observed in later follow-up, such as rapid declines or jumps, likely reflect statistical instability due to the small number of participants still at risk. Fourth, we acknowledge that multiple comparisons were made across several carotenoids measures and mortality outcomes, which may increase the risk of type I error. Finally, as an observational cohort study, although we adjusted for multiple covariates, residual confounding from factors such as changes in dietary habits, smoking, oral hygiene practices, or socioeconomic variables during follow-up cannot be completely ruled out; therefore, causal inference in this study is limited.

## Conclusion

Higher serum levels of selected carotenoids were associated with a weaker relationship between serum *P. gingivalis* IgG levels and all-cause, CeVD, and DM mortality. These findings suggest that carotenoids-related nutritional status may play a modifying role in the association between periodontal pathogen exposure and long-term mortality.

## Supplementary Information

Additional file 1: Figure S1. The flowchart of this study. Figure S2. The survival probability of specific cause mortality by serum *P. gingivalis* IgG groups. (A) CVD mortality; (B) Cancer mortality; (C) CeVD mortality; (D) DM mortality Abbreviation:*P. gingivalis*, Porphyromonas gingivalis; IgG, immunoglobulin G; CI, confidence interval; CVD, cardiovascular disease; CeVD, cerebrovascular disease; DM, diabetes mellitus. Figure S3. The dose-response associations between serum *P. gingivalis* IgG levels and specific cause mortality. (A) CVD mortality; (B) Cancer mortality; (C) CeVD mortality; (D) DM mortalit. Abbreviation: *P. gingivalis*, Porphyromonas gingivalis; HR, hazard ratio; CI, confidence interval; IgG, immunoglobulin G; CVD, cardiovascular disease; CeVD, cerebrovascular disease; DM, diabetes mellitus. The restricted cubic spline models were adjusted by sex (males or females), age (20-39, 40-59,≥60 years), race-ethnicity (non-Hispanic white, non-Hispanic black, Mexican American and other), education (less than junior high school, junior high school, high school, college or above), income (<$20,000, ≥$20,000), smoking status (current smokers, former smokers, never smokers), drinking status (≥12 drinks in the past 12 months,<12 drinks in the past 12 months), exercise (more active, less active, about the same), body mass index (<18.5, 18.5-24.9, 25-29.9, ≥30kg/m^2^). Figure S4. The interactions between serum carotenoids and serum *P. gingivalis* IgG levels on cardiovascular disease mortality. Abbreviation: *P. gingivalis*, Porphyromonas gingivalis; IgG, immunoglobulin G; CI, confidence interval; CVD, cardiovascular disease. The cox proportional hazard models were adjusted by sex (males or females), age (20-39, 40-59, ≥60 years), race-ethnicity (non-Hispanic white, non-Hispanic black, Mexican American and other), education (less than junior high school, junior high school, high school, college or above), income (<$20,000, ≥$20,000), smoking status (current smokers, former smokers, never smokers), drinking status (≥12 drinks in the past 12 months,<12 drinks in the past 12 months), exercise (more active, less active, about the same), body mass index (<18.5, 18.5-24.9, 25-29.9, ≥30kg/m^2^). Figure S5. The interactions between serum carotenoids and serum *P. gingivalis* IgG levels on cancer mortality. Abbreviation: *P. gingivalis*, Porphyromonas gingivalis; IgG, immunoglobulin G; CI, confidence interval. The cox proportional hazard models were adjusted by sex (males or females), age (20-39, 40-59, ≥60 years), race-ethnicity (non-Hispanic white, non-Hispanic black, Mexican American and other), education (less than junior high school, junior high school, high school, college or above), income (<$20,000, ≥$20,000), smoking status (current smokers, former smokers, never smokers), drinking status (≥12 drinks in the past 12 months,<12 drinks in the past 12 months), exercise (more active, less active, about the same), body mass index (<18.5, 18.5-24.9, 25-29.9, ≥30kg/m^2^). Figure S6. The interactions between serum carotenoids and serum *P. gingivalis* IgG levels on cerebrovascular disease mortality. Abbreviation:*P. gingivalis*, Porphyromonas gingivalis; IgG, immunoglobulin G; CI, confidence interval; CeVD, cerebrovascular disease. The cox proportional hazard models were adjusted by sex (males or females), age (20-39, 40-59, ≥60 years), race-ethnicity (non-Hispanic white, non-Hispanic black, Mexican American and other), education (less than junior high school, junior high school, high school, college or above), income (<$20,000, ≥$20,000), smoking status (current smokers, former smokers, never smokers), drinking status (≥12 drinks in the past 12 months, <12 drinks in the past 12 months), exercise (more active, less active, about the same), body mass index (<18.5, 18.5-24.9, 25-29.9, ≥30kg/m^2^). Figure S7. The interactions between serum carotenoids and serum *P. gingivalis* IgG levels on diabetes mellitus mortality. Abbreviation:*P. gingivalis*, Porphyromonas gingivalis; IgG, immunoglobulin G; CI, confidence interval; DM, diabetes mellitus. The cox proportional hazard models were adjusted by sex (males or females), age (20-39, 40-59, ≥60 years), race-ethnicity (non-Hispanic white, non-Hispanic black, Mexican American and other), education (less than junior high school, junior high school, high school, college or above), income (<$20,000, ≥$20,000), smoking status (current smokers, former smokers, never smokers), drinking status (≥12 drinks in the past 12 months,<12 drinks in the past 12 months), exercise (more active, less active, about the same), body mass index (<18.5, 18.5-24.9, 25-29.9, ≥30kg/m^2^). Figure S8. The joint associations of serum carotenoids and serum *P. gingivalis* IgG levels on specific-cause mortality. Abbreviation: *P. gingivalis*, Porphyromonas gingivalis; IgG, immunoglobulin G; CI, confidence interval. The cox proportional hazard models were adjusted by sex (males or females), age (20-39, 40-59, ≥60 years), race-ethnicity (non-Hispanic white, non-Hispanic black, Mexican American and other), education (less than junior high school, junior high school, high school, college or above), income (<$20,000, ≥$20,000), smoking status (current smokers, former smokers, never smokers), drinking status (≥12 drinks in the past 12 months,<12 drinks in the past 12 months), exercise (more active, less active, about the same), body mass index (<18.5, 18.5-24.9, 25-29.9, ≥30kg/m^2^).

Additional file 2.

## Data Availability

The datasets supporting the conclusions of this article are available in the NHANES repository, https://wwwn.cdc.gov/nchs/nhanes/Default.aspx.

## References

[1] Baker JL, Mark Welch JL, Kauffman KM, McLean JS, He X. The oral microbiome: diversity, biogeography and human health. Nat Rev Microbiol. 2024;22(2):89–104. 10.1038/s41579-023-00963-6. PMC11084736 37700024

[2] Reyes L. Porphyromonas gingivalis. Trends Microbiol. 2021;29(4):376–7. 10.1016/j.tim.2021.01.010. 33546976

[3] Hajishengallis G, Darveau RP, Curtis MA. The keystone-pathogen hypothesis. Nat Rev Microbiol. 2012;10(10):717–25. 10.1038/nrmicro2873. PMC3498498 22941505

[4] Vlachojannis C, Dye BA, Herrera-Abreu M, et al. Determinants of serum IgG responses to periodontal bacteria in a nationally representative sample of US adults. J Clin Periodontol. 2010;37(8):685–96. 10.1111/j.1600-051X.2010.01592.x. 20561113

[5] Lakio L, Antinheimo J, Paju S, Buhlin K, Pussinen PJ, Alfthan G. Tracking of plasma antibodies against *Aggregatibacter actinomycetemcomitans* and *Porphyromonas gingivalis* during 15 years. J Oral Microbiol. 2009. 10.3402/jom.v1i0.1979. PMC3077000 21523211

[6] Merchant AT, Shrestha D, Chaisson C, Choi YH, Hazlett LJ, Zhang J. Association between serum antibodies to oral microorganisms and hyperglycemia in adults. J Dent Res. 2014;93(8):752–9. 10.1177/0022034514538451. PMC4293758 24943202

[7] Sanchez-Torres D, Gutierrez-Bejarano D, Hurtado-Roca Y, Guallar-Castillon P, Muntner P, Laclaustra M. Non-linear association of periodontal pathogen antibodies with mortality. Int J Cardiol. 2015;187:628–36. 10.1016/j.ijcard.2015.03.315. 25863738

[8] Joshi C, Bapat R, Anderson W, Dawson D, Cherukara G, Hijazi K. Serum antibody response against periodontal bacteria and coronary heart disease: systematic review and meta-analysis. J Clin Periodontol. 2021;48(12):1570–86. 10.1111/jcpe.13550. 34545592

[9] Bohnstedt S, Cullinan MP, Ford PJ, et al. High antibody levels to P. gingivalis in cardiovascular disease. J Dent Res. 2010;89(9):938–42. 10.1177/0022034510370817 20519492

[10] Kataoka A, Katagiri S, Kawashima H, et al. Association between periodontal bacteria and degenerative aortic stenosis: a pilot study. J Periodontal Implant Sci. 2021;51(4):226–38. 10.5051/jpis.2006040302. PMC8367646 34387043

[11] Svard A, Kastbom A, Ljungberg KR, et al. Antibodies against *Porphyromonas gingivalis* in serum and saliva and their association with rheumatoid arthritis and periodontitis. Data from two rheumatoid arthritis cohorts in Sweden. Front Immunol. 2023;14:1183194. 10.3389/fimmu.2023.1183194. PMC10265683 37325636

[12] Iwasaki M, Taylor GW, Manz MC, et al. Serum antibody to *Porphyromonas gingivalis* in chronic kidney disease. J Dent Res. 2012;91(9):828–33. 10.1177/0022034512455063. 22828790

[13] Liu S, Butler CA, Ayton S, Reynolds EC, Dashper SG. Porphyromonas gingivalis and the pathogenesis of Alzheimer's disease. Crit Rev Microbiol. 2023:1–11. 10.1080/1040841X.2022.2163613 36597758

[14] Huang Y, Wei Y, Xu J, Wei X. A comprehensive review on the prevention and regulation of Alzheimer's disease by tea and its active ingredients. Critical reviews in food science and nutrition. 2022:1–25. 10.1080/10408398.2022.2081128 35647742

[15] Ahn J, Segers S, Hayes RB. Periodontal disease, *Porphyromonas gingivalis* serum antibody levels and orodigestive cancer mortality. Carcinogenesis. 2012;33(5):1055–8. 10.1093/carcin/bgs112. PMC3334514 22367402

[16] Zhou B, Lu J, Beck JD, et al. Periodontal and other oral bacteria and risk of lung cancer in the Atherosclerosis Risk in Communities (ARIC) Study. Cancer Epidemiol Biomarkers Prev. 2023;32(4):505–15. 10.1158/1055-9965.EPI-22-0601. PMC9947191 35999656

[17] Rodriguez-Concepcion M, Avalos J, Bonet ML, et al. A global perspective on carotenoids: metabolism, biotechnology, and benefits for nutrition and health. Prog Lipid Res. 2018;70:62–93. 10.1016/j.plipres.2018.04.004. 29679619

[18] Maiani G, Caston MJ, Catasta G, et al. Carotenoids: actual knowledge on food sources, intakes, stability and bioavailability and their protective role in humans. Mol Nutr Food Res. 2009;53(Suppl 2):S194-218. 10.1002/mnfr.200800053. 19035552

[19] Kaur G, Kathariya R, Bansal S, Singh A, Shahakar D. Dietary antioxidants and their indispensable role in periodontal health. J Food Drug Anal. 2016;24(2):239–46. 10.1016/j.jfda.2015.11.003. PMC9339570 28911576

[20] Naruishi K. Carotenoids and periodontal infection. Nutrients. 2020. 10.3390/nu12010269. PMC7019381 31968635

[21] Burt VL, Harris T. The third National Health and Nutrition Examination Survey: contributing data on aging and health. Gerontologist. 1994;34(4):486–90. 10.1093/geront/34.4.486. 7959106

[22] Dye BA, Herrera-Abreu M, Lerche-Sehm J, et al. Serum antibodies to periodontal bacteria as diagnostic markers of periodontitis. J Periodontol. 2009;80(4):634–47. 10.1902/jop.2009.080474. 19335084

[23] Offenbacher S, Barros SP, Singer RE, Moss K, Williams RC, Beck JD. Periodontal disease at the biofilm-gingival interface. J Periodontol. 2007;78(10):1911–25. 10.1902/jop.2007.060465. 18062113

[24] National Health and Nutrition Examination Survey Codebook for Data Production (1991–1994) (NHANES III) Periodontal Pathogens: Serum Actinobacillus actinomycetemcomitans Antibody and Porphyromonas gingivalis Antibodies http://www.cdc.gov/nchs/data/nhanes/nhanes3/depp.pdf.

[25] Gunter EW, Lewis BG, Koncikowski SM. Laboratory procedures used for the third National Health and Nutrition Examination Survey (NHANES III), 1988–1994.1996.Accessed 16 August 2023. Available from https://stacks.cdc.gov/view/cdc/45776.

[26] Centers for Disease Control and Prevention. National Center for Health Statistics (NCHS). National Health and Nutrition Examination Survey Questionnaire (or Examination Protocol,orLaboratory Protocol).2006. Accessed 16 August 2023. Available from https://www.cdcgov/nchs/nhanes.htm.

[27] Li C, Yu R, Ding Y. Association between *Porphyromonas gingivalis* and systemic diseases: focus on T cells-mediated adaptive immunity. Front Cell Infect Microbiol. 2022;12:1026457. 10.3389/fcimb.2022.1026457. PMC9712990 36467726

[28] Ruan Q, Guan P, Qi W, et al. *Porphyromonas gingivalis* regulates atherosclerosis through an immune pathway. Front Immunol. 2023;14:1103592. 10.3389/fimmu.2023.1103592. PMC10043234 36999040

[29] Kong J, Liu Y, Qian M, Xing L, Gao S. The relationship between *Porphyromonas gingivalis* and oesophageal squamous cell carcinoma: a literature review. Epidemiol Infect. 2023;151:e69. 10.1017/S0950268823000298. PMC10204138 37009715

[30] Jia S, Li X, Du Q. Host insulin resistance caused by *Porphyromonas gingivalis*-review of recent progresses. Front Cell Infect Microbiol. 2023;13:1209381. 10.3389/fcimb.2023.1209381. PMC10373507 37520442

[31] Bohm V, Lietz G, Olmedilla-Alonso B, et al. From carotenoid intake to carotenoid blood and tissue concentrations - implications for dietary intake recommendations. Nutr Rev. 2021;79(5):544–73. 10.1093/nutrit/nuaa008. PMC8025354 32766681

[32] Terao J. Revisiting carotenoids as dietary antioxidants for human health and disease prevention. Food Funct. 2023;14(17):7799–824. 10.1039/d3fo02330c. 37593767

[33] Tapiero H, Townsend DM, Tew KD. The role of carotenoids in the prevention of human pathologies. Biomed Pharmacother. 2004;58(2):100–10. 10.1016/j.biopha.2003.12.006. PMC6361147 14992791

[34] Ba W, Xu W, Deng Z, Zhang B, Zheng L, Li H. The Antioxidant and Anti-Inflammatory Effects of the Main Carotenoids from Tomatoes via Nrf2 and NF-kappaB Signaling Pathways. Nutrients. 2023;15(21) 10.3390/nu15214652 PMC10650085 37960305

[35] Kose O, Arabaci T, Yemenoglu H, et al. Influences of fucoxanthin on alveolar bone resorption in induced periodontitis in rat molars. Mar Drugs. 2016. 10.3390/md14040070. PMC4849074 27043583

[36] Figgins EL, Arora P, Gao D, et al. Enhancement of innate immunity in gingival epithelial cells by vitamin D and HDAC inhibitors. Front Oral Health. 2024;5:1378566. 10.3389/froh.2024.1378566. PMC10986367 38567313

[37] Moszura J, Gawlak-Socka S, Peksa J, Bielecka-Kowalska N, Klosek S. The Role of Vitamin D(3) in Periodontal Health: Implications for Bone Metabolism, Immune Modulation and Inflammation Control. Nutrients. 2026;18(4) 10.3390/nu18040577 PMC12943040 41754094

[38] Meghil MM, Cutler CW. Influence of vitamin D on periodontal inflammation: a review. Pathogens. 2023. 10.3390/pathogens12091180. PMC10537363 37764988

[39] Niu L, Chen S, Yang X, et al. Vitamin D decreases *Porphyromonas gingivalis* internalized into macrophages by promoting autophagy. Oral Dis. 2021;27(7):1775–88. 10.1111/odi.13696. 33098722

[40] Kajiura Y, Nishikawa Y, Lew JH, Kido JI, Nagata T, Naruishi K. beta-carotene suppresses Porphyromonas gingivalis lipopolysaccharide-mediated cytokine production in THP-1 monocytes cultured with high glucose condition. Cell Biol Int. 2018;42(1):105–11. 10.1002/cbin.10873. 28906038

[41] Clarke R, Shipley M, Lewington S, et al. Underestimation of risk associations due to regression dilution in long-term follow-up of prospective studies. Am J Epidemiol. 1999;150(4):341–53. 10.1093/oxfordjournals.aje.a010013. 10453810

