# Supplementary material for: Serum carotenoids modify the association between porphyromonas gingivalis IgG and mortality: a prospective cohort study with 25.8 years of follow-up
Source: BMC Nutr. 2026 Jul 6;12:202. doi: 10.1186/s40795-026-01420-2 (PMC13617849; doi:10.1186/s40795-026-01420-2)
Supplement: Supplementary file 1 [file 40795_2026_1420_MOESM1_ESM.docx]

Supplementary Material

## Supplementary Figures

**Supplementary Figure 1.** The flowchart of this study.

**Supplementary Figure 2. The survival probability of specific cause mortality by serum *P. gingivalis* IgG groups.**

(A) CVD mortality; (B) Cancer mortality; (C) CeVD mortality; (D) DM mortality

Abbreviation: *P. gingivalis*, Porphyromonas gingivalis; IgG, immunoglobulin G; CI, confidence interval; CVD, cardiovascular disease; CeVD, cerebrovascular disease; DM, diabetes mellitus.

**Supplementary Figure 3. The dose-response associations between serum *P. gingivalis* IgG levels and specific cause mortality.**

(A) CVD mortality; (B) Cancer mortality; (C) CeVD mortality; (D) DM mortality

Abbreviation: *P. gingivalis*, Porphyromonas gingivalis; HR, hazard ratio; CI, confidence interval; IgG, immunoglobulin G; CVD, cardiovascular disease; CeVD, cerebrovascular disease; DM, diabetes mellitus.

The restricted cubic spline models were adjusted by sex (males or females), age (20-39, 40-59, ≥60 years), race-ethnicity (non-Hispanic white, non-Hispanic black, Mexican American and other), education (less than junior high school, junior high school, high school, college or above), income (<$20,000, ≥$20,000), smoking status (current smokers, former smokers, never smokers), drinking status (≥12 drinks in the past 12 months, <12 drinks in the past 12 months), exercise (more active, less active, about the same), body mass index (<18.5, 18.5-24.9, 25-29.9, ≥30kg/m^2^).

**Supplementary Figure 4. The interactions between serum carotenoids and serum *P. gingivalis* IgG levels on cardiovascular disease mortality.**

Abbreviation: *P. gingivalis*, Porphyromonas gingivalis; IgG, immunoglobulin G; CI, confidence interval; CVD, cardiovascular disease.

The cox proportional hazard models were adjusted by sex (males or females), age (20-39, 40-59, ≥60 years), race-ethnicity (non-Hispanic white, non-Hispanic black, Mexican American and other), education (less than junior high school, junior high school, high school, college or above), income (<$20,000, ≥$20,000), smoking status (current smokers, former smokers, never smokers), drinking status (≥12 drinks in the past 12 months, <12 drinks in the past 12 months), exercise (more active, less active, about the same), body mass index (<18.5, 18.5-24.9, 25-29.9, ≥30kg/m^2^).

**Supplementary Figure 5.** **The interactions between serum carotenoids and serum *P. gingivalis* IgG levels on cancer mortality.**

Abbreviation: *P. gingivalis*, Porphyromonas gingivalis; IgG, immunoglobulin G; CI, confidence interval.

The cox proportional hazard models were adjusted by sex (males or females), age (20-39, 40-59, ≥60 years), race-ethnicity (non-Hispanic white, non-Hispanic black, Mexican American and other), education (less than junior high school, junior high school, high school, college or above), income (<$20,000, ≥$20,000), smoking status (current smokers, former smokers, never smokers), drinking status (≥12 drinks in the past 12 months, <12 drinks in the past 12 months), exercise (more active, less active, about the same), body mass index (<18.5, 18.5-24.9, 25-29.9, ≥30kg/m^2^).

**Supplementary Figure 6.** **The interactions between serum carotenoids and serum *P. gingivalis* IgG levels on cerebrovascular disease mortality.**

Abbreviation: *P. gingivalis*, Porphyromonas gingivalis; IgG, immunoglobulin G; CI, confidence interval; CeVD, cerebrovascular disease.

The cox proportional hazard models were adjusted by sex (males or females), age (20-39, 40-59, ≥60 years), race-ethnicity (non-Hispanic white, non-Hispanic black, Mexican American and other), education (less than junior high school, junior high school, high school, college or above), income (<$20,000, ≥$20,000), smoking status (current smokers, former smokers, never smokers), drinking status (≥12 drinks in the past 12 months, <12 drinks in the past 12 months), exercise (more active, less active, about the same), body mass index (<18.5, 18.5-24.9, 25-29.9, ≥30kg/m^2^).

**Supplementary Figure 7. The interactions between serum carotenoids and serum *P. gingivalis* IgG levels on diabetes mellitus mortality.**

Abbreviation: *P. gingivalis*, Porphyromonas gingivalis; IgG, immunoglobulin G; CI, confidence interval; DM, diabetes mellitus.

The cox proportional hazard models were adjusted by sex (males or females), age (20-39, 40-59, ≥60 years), race-ethnicity (non-Hispanic white, non-Hispanic black, Mexican American and other), education (less than junior high school, junior high school, high school, college or above), income (<$20,000, ≥$20,000), smoking status (current smokers, former smokers, never smokers), drinking status (≥12 drinks in the past 12 months, <12 drinks in the past 12 months), exercise (more active, less active, about the same), body mass index (<18.5, 18.5-24.9, 25-29.9, ≥30kg/m^2^).

**Figure S8. The joint associations of serum carotenoids and serum *P. gingivalis* IgG levels on specific-cause mortality.**

Abbreviation: P. gingivalis, Porphyromonas gingivalis; IgG, immunoglobulin G; CI, confidence interval.

The cox proportional hazard models were adjusted by sex (males or females), age (20-39, 40-59, ≥60 years), race-ethnicity (non-Hispanic white, non-Hispanic black, Mexican American and other), education (less than junior high school, junior high school, high school, college or above), income (<$20,000, ≥$20,000), smoking status (current smokers, former smokers, never smokers), drinking status (≥12 drinks in the past 12 months, <12 drinks in the past 12 months), exercise (more active, less active, about the same), body mass index (<18.5, 18.5-24.9, 25-29.9, ≥30kg/m^2^).
