# Supplementary material for: Serum carotenoids modify the association between porphyromonas gingivalis IgG and mortality: a prospective cohort study with 25.8 years of follow-up
Source: BMC Nutr. 2026 Jul 6;12:202. doi: 10.1186/s40795-026-01420-2 (PMC13617849; doi:10.1186/s40795-026-01420-2)
Supplement: Supplementary file 2 [file 40795_2026_1420_MOESM2_ESM.docx]

| **Table S1.Hazard ratios (95% CIs) of mortality in relation to serum** Porphyromonas gingivalis IgG. | | | | | | | | | | |
| --- | --- | --- | --- | --- | --- | --- | --- | --- | --- | --- |
|  | | | Serum Porphyromonas gingivalis IgG | | | | | | |  |
|  | | | <71 EU | 71-101 EU | | >101 EU | | *P* for trend | | 71 + EU |
| **All-cause mortality** | | |  |  | |  | |  | |  |
| Age and gender-adjusted HR (95% CI) | | | 1.00 (ref) | 1.16 (1.09-1.23) | | 1.17 (1.05-1.29) | | 0.004 | | 1.16 (1.09-1.24) |
| Multivariable-adjusted HR (95% CI)^*^ | | | 1.00 (ref) | 1.09 (1.04-1.16) | | 1.14 (1.05-1.23) | | 0.003 | | 1.11 (1.05-1.17) |
| **CVD mortality** | | |  |  | |  | |  | |  |
| Age and gender-adjusted HR (95% CI) | | | 1.00 (ref) | 1.33 (1.17-1.51) | | 1.31 (1.13-1.53) | | 0.003 | | 1.32 (1.16-1.50) |
| Multivariable-adjusted HR (95% CI)^*^ | | | 1.00 (ref) | 1.31 (1.14-1.49) | | 1.32 (1.14-1.54) | | 0.001 | | 1.31 (1.18-1.46) |
| **Cancer mortality** | | |  |  | |  | |  | |  |
| Age and gender-adjusted HR (95% CI) | | | 1.00 (ref) | 0.96 (0.85-1.09) | | 1.06 (0.90-1.24) | | 0.47 | | 1.01 (0.93-1.08) |
| Multivariable-adjusted HR (95% CI)^*^ | | | 1.00 (ref) | 0.97 (0.82-1.15) | | 1.03 (0.90-1.19) | | 0.71 | | 1.00 (0.87-1.14) |
| **Ce**VD mortality | | |  |  | |  | |  | |  |
| Age and gender-adjusted HR (95% CI) | | | 1.00 (ref) | 1.34 (1.17-1.55) | | 1.27 (1.00-1.63) | | 0.04 | | 1.31 (1.10-1.57) |
| Multivariable-adjusted HR (95% CI)^*^ | | | 1.00 (ref) | 1.46 (1.26-1.70) | | 1.39 (1.10-1.77) | | 0.007 | | 1.44 (1.20-1.71) |
| **D**M mortality | | |  |  | |  | |  | |  |
| Age and gender-adjusted HR (95% CI) | | | 1.00 (ref) | 2.35 (2.20-2.51) | | 1.99 (1.69-2.35) | | <0.001 | | 2.20 (2.02-2.40) |
| Multivariable-adjusted HR (95% CI)^*^ | | | 1.00 (ref) | 2.47 (2.25-2.71) | | 1.94 (1.68-2.24) | | <0.001 | | 2.27 (2.07-2.48) |
| Abbreviations: HR, hazard ratio; CI, confidence interval; CVD, cardiovascular disease; CeVD, cerebrovascular disease; DM, diabetes mellitus. | | | | | | | | | | |
| ^*^The cox proportional hazard models were adjusted by sex (males or females), age (20-39,40-59,≥60 years), race-ethnicity (non-Hispanic white, non-Hispanic black, Mexican American and other), education (less than junior high school, junior high school, high school, college or above), income (<$20,000, ≥$20,000), smoking status (current smokers, former smokers, never smokers), drinking status (≥12 drinks in the past 12 months, <12 drinks in the past 12 months), exercise (more active, less active, about the same), body mass index (<18.5, 18.5-24.9, 25-29.9, ≥30kg/m^2^). | | | | | | | | | | |
| **Table S2.The interactions between serum carotenoids and serum** P. gingivalis **IgG levels on mortality** | | | | | | | | |  |  |
| **Serum** **carotenoids** | | **HR (95% CI) of *P. gingivalis* IgG (71+EU vs. <71EU)** | | | | | ***P***_int_ | |  |  |
|  |  | Lower carotenoids | | | Higher carotenoids | |  |  |  |  |
| **All-cause mortality** | | | | | | | | |  |  |
|  | α-carotene | 1.16 (1.10-1.22) | | | 1.09 (1.03-1.16) | | 0.09 | |  |  |
|  | β-carotene | 1.19 (1.08-1.32) | | | 1.06 (0.98-1.15) | | 0.24 | |  |  |
|  | β-cryptoxanthin | 1.12 (1.07-1.17) | | | 1.08 (0.99-1.17) | | 0.96 | |  |  |
|  | lutein/zeaxanthin | 1.16 (1.06-1.27) | | | 1.08 (1.00-1.17) | | 0.31 | |  |  |
|  | lycopene | 1.08 (0.98-1.19) | | | 1.08 (1.00-1.17) | | 0.95 | |  |  |
| **CVD mortality** | | | | | | | | |  |  |
|  | α-carotene | 1.22 (0.99-1.50) | | | 1.37 (1.22-1.54) | | 0.14 | |  |  |
|  | β-carotene | 1.08 (0.81-1.44) | | | 1.46 (1.34-1.59) | | 0.02 | |  |  |
|  | β-cryptoxanthin | 1.23 (1.02-1.50) | | | 1.31 (1.11-1.55) | | 0.30 | |  |  |
|  | lutein/zeaxanthin | 1.38 (1.11-1.70) | | | 1.37 (1.28-1.46) | | 0.55 | |  |  |
|  | lycopene | 1.17 (1.02-1.35) | | | 1.26 (1.08-1.47) | | 0.004 | |  |  |
| **Cancer mortality** | | | | | | | | |  |  |
|  | α-carotene | 0.86 (0.77-0.96) | | | 1.20 (0.97-1.49) | | 0.001 | |  |  |
|  | β-carotene | 0.81 (0.70-0.93) | | | 1.13 (0.83-1.52) | | 0.02 | |  |  |
|  | β-cryptoxanthin | 0.91 (0.85-0.98) | | | 1.10 (0.76-1.61) | | 0.03 | |  |  |
|  | lutein/zeaxanthin | 0.91 (0.79-1.03) | | | 1.11 (0.80-1.53) | | 0.10 | |  |  |
|  | lycopene | 1.03 (0.91-1.17) | | | 0.92 (0.70-1.23) | | 0.52 | |  |  |
| **Ce**VD mortality | | | | | | | | |  |  |
|  | α-carotene | 1.92 (1.46-2.54) | | | 1.16 (0.91-1.48) | | 0.007 | |  |  |
|  | β-carotene | 1.79 (1.41-2.26) | | | 1.27 (1.02-1.58) | | 0.09 | |  |  |
|  | β-cryptoxanthin | 0.98 (0.90-1.07) | | | 1.74 (1.38-2.20) | | 0.053 | |  |  |
|  | lutein/zeaxanthin | 1.60 (1.33-1.92) | | | 1.14 (0.92-1.43) | | <0.001 | |  |  |
|  | lycopene | 1.90 (1.41-2.54) | | | 0.82 (0.63-1.07) | | <0.001 | |  |  |
| **D**M mortality | | | | | | | | |  |  |
|  | α-carotene | 3.87 (3.30-4.54) | | | 1.28 (1.05-1.55) | | <0.001 | |  |  |
|  | β-carotene | 3.02 (2.33-3.92) | | | 0.98 (0.80-1.21) | | 0.001 | |  |  |
|  | β-cryptoxanthin | 4.36 (3.67-5.17) | | | 0.94 (0.76-1.17) | | <0.001 | |  |  |
|  | lutein/zeaxanthin | 3.91 (2.79-5.47) | | | 0.94 (0.78-1.15) | | <0.001 | |  |  |
|  | lycopene | 3.40 (2.35-4.90) | | | 0.66 (0.57-0.75) | | <0.001 | |  |  |
| Abbreviations: HR, hazard ratio; CI, confidence interval; CVD, cardiovascular disease; CeVD, cerebrovascular disease; DM, diabetes mellitus. | | | | | | | | |  |  |
| The cox proportional hazard models were adjusted by sex (males or females), age (20-39,40-59,≥60 years), race-ethnicity (non-Hispanic white, non-Hispanic black, Mexican American and other), education (less than junior high school, junior high school, high school, college or above), income (<$20,000, ≥$20,000), smoking status (current smokers, former smokers, never smokers), drinking status (≥12 drinks in the past 12 months, <12 drinks in the past 12 months), exercise (more active, less active, about the same), body mass index (<18.5, 18.5-24.9, 25-29.9, ≥30kg/m^2^). | | | | | | | | |  |  |

| **Table S3. The joint associations of serum carotenoids and *P. gingivalis* IgG levels on mortality** | | | | | | |
| --- | --- | --- | --- | --- | --- | --- |
|  | | HR (95% CI) | | | | *P* trend |
|  |  | *P. gingivalis* IgG ≥ 71/lower carotenoids | *P. gingivalis* IgG < 71/lower carotenoids | *P. gingivalis* IgG ≥ 71/higher carotenoids | *P. gingivalis* IgG < 71/higher carotenoids |  |
| **All-cause mortality** | |  |  |  |  |  |
|  | α-carotene | ref | 0.88 (0.81-0.95) | 0.78 (0.73-0.83) | 0.73 (0.67-0.79) | <0.001 |
|  | β-carotene | ref | 0.86 (0.74-1.00) | 0.82 (0.74-0.91) | 0.78 (0.71-0.85) | <0.001 |
|  | β-cryptoxanthin | ref | 0.92 (0.88-0.95) | 0.76 (0.68-0.84) | 0.69 (0.64-0.75) | <0.001 |
|  | lutein/zeaxanthin | ref | 0.88 (0.78-0.99) | 0.83 (0.74-0.92) | 0.77 (0.70-0.85) | <0.001 |
|  | lycopene | ref | 0.91 (0.81-1.03) | 0.69 (0.61-0.79) | 0.64 (0.60-0.68) | <0.001 |
| **CVD mortality** | |  |  |  |  |  |
|  | α-carotene | ref | 0.86 (0.69-1.06) | 0.85 (0.76-0.96) | 0.61 (0.55-0.68) | <0.001 |
|  | β-carotene | ref | 0.97 (0.74-1.25) | 1.05 (0.93-1.17) | 0.70 (0.61-0.81) | 0.001 |
|  | β-cryptoxanthin | ref | 0.81 (0.69-0.97) | 0.88 (0.78-1.00) | 0.63 (0.55-0.72) | <0.001 |
|  | lutein/zeaxanthin | ref | 0.78 (0.65-0.93) | 1.04 (0.94-1.15) | 0.78 (0.69-0.87) | 0.01 |
|  | lycopene | ref | 0.85 (0.74-0.96) | 0.89 (0.79-0.99) | 0.62 (0.52-0.74) | <0.001 |
| **Cancer mortality** | |  |  |  |  |  |
|  | α-carotene | ref | 1.17 (1.04-1.32) | 0.84 (0.72-0.99) | 0.75 (0.68-0.82) | <0.001 |
|  | β-carotene | ref | 1.20 (1.07-1.34) | 0.83 (0.69-1.01) | 0.74 (0.66-0.84) | <0.001 |
|  | β-cryptoxanthin | ref | 1.11 (1.02-1.21) | 0.70 (0.53-0.92) | 0.60 (0.53-0.68) | <0.001 |
|  | lutein/zeaxanthin | ref | 1.11 (1.01-1.22) | 0.94 (0.77-1.13) | 0.85 (0.76-0.94) | <0.001 |
|  | lycopene | ref | 1.09 (0.97-1.23) | 0.70 (0.61-0.82) | 0.70 (0.58-0.84) | <0.001 |
| **CeVD mortality** | |  |  |  |  |  |
|  | α-carotene | ref | 0.60 (0.49-0.75) | 0.44 (0.38-0.51) | 0.39 (0.34-0.45) | <0.001 |
|  | β-carotene | ref | 0.62 (0.46-0.85) | 0.52 (0.44-0.62) | 0.42 (0.36-0.50) | <0.001 |
|  | β-cryptoxanthin | ref | 0.78 (0.70-0.88) | 0.68 (0.54-0.84) | 0.45 (0.39-0.52) | <0.001 |
|  | lutein/zeaxanthin | ref | 0.53 (0.44-0.64) | 0.77 (0.68-0.86) | 0.66 (0.54-0.8) | 0.001 |
|  | lycopene | ref | 0.52 (0.39-0.69) | 0.37 (0.30-0.46) | 0.44 (0.37-0.52) | <0.001 |
| **DM mortality** | |  |  |  |  |  |
|  | α-carotene | ref | 0.34 (0.29-0.39) | 0.48 (0.40-0.59) | 0.32 (0.27-0.37) | <0.001 |
|  | β-carotene | ref | 0.37 (0.3-0.46) | 0.38 (0.32-0.45) | 0.26 (0.24-0.29) | <0.001 |
|  | β-cryptoxanthin | ref | 0.30 (0.26-0.34) | 0.60 (0.52-0.70) | 0.48 (0.41-0.57) | <0.001 |
|  | lutein/zeaxanthin | ref | 0.30 (0.25-0.36) | 0.35 (0.31-0.41) | 0.32 (0.28-0.36) | <0.001 |
|  | lycopene | ref | 0.29 (0.23-0.37) | 0.18 (0.16-0.22) | 0.20 (0.17-0.22) | <0.001 |
| Abbreviations: HR, hazard ratio; CI, confidence interval; CVD, cardiovascular disease; CeVD, cerebrovascular disease; DM, diabetes mellitus. | | | | | | |
| The cox proportional hazard models were adjusted by sex (males or females), age (20-39,40-59,≥60 years), race-ethnicity (non-Hispanic white, non-Hispanic black, Mexican American and other), education (less than junior high school, junior high school, high school, college or above), income (<$20,000, ≥$20,000), smoking status (current smokers, former smokers, never smokers), drinking status (≥12 drinks in the past 12 months, <12 drinks in the past 12 months), exercise (more active, less active, about the same), body mass index (<18.5, 18.5-24.9, 25-29.9, ≥30kg/m^2^). | | | | | | |
